# Rifampicin Induces Gene, Protein, and Activity of P-Glycoprotein (ABCB1) in Human Precision-Cut Intestinal Slices

**DOI:** 10.3389/fphar.2021.684156

**Published:** 2021-06-09

**Authors:** Ondrej Martinec, Carin Biel, Inge A. M. de Graaf, Martin Huliciak, Koert P. de Jong, Frantisek Staud, Filip Cecka, Peter Olinga, Ivan Vokral, Lukas Cerveny

**Affiliations:** ^1^ Department of Pharmacology and Toxicology, Faculty of Pharmacy in Hradec Kralove, Charles University, Hradec Kralove, Czechia; ^2^ Department of Medical Biochemistry, Faculty of Medicine in Hradec Kralove, Charles University, Hradec Kralove, Czechia; ^3^ Department of Pharmaceutical Technology and Biopharmacy, Faculty of Science and Engineering, Groningen Research Institute of Pharmacy, Groningen, Netherlands; ^4^ Graduate School of Science, Faculty of Science and Engineering, University of Groningen, Groningen, Netherlands; ^5^ Department of Hepato-Pancreato-Biliary Surgery and Liver Transplantation, University Medical Center Groningen, University of Groningen, Groningen, Netherlands; ^6^ Department of Surgery, University Hospital Hradec Kralove, Hradec Kralove, Czechia

**Keywords:** induction, P-glycoprotein (ABCB1 protein), human precision-cut intestinal slices, rifampicin, absorption, pregnane X receptor

## Abstract

P-glycoprotein (ABCB1), an ATP-binding cassette efflux transporter, limits intestinal absorption of its substrates and is a common site of drug–drug interactions. Drug-mediated induction of intestinal ABCB1 is a clinically relevant phenomenon associated with significantly decreased drug bioavailability. Currently, there are no well-established human models for evaluating its induction, so drug regulatory authorities provide no recommendations for *in vitro*/*ex vivo* testing drugs’ ABCB1-inducing activity. Human precision-cut intestinal slices (hPCISs) contain cells in their natural environment and express physiological levels of nuclear factors required for ABCB1 induction. We found that hPCISs incubated in William’s Medium E for 48 h maintained intact morphology, ATP content, and ABCB1 efflux activity. Here, we asked whether rifampicin (a model ligand of pregnane X receptor, PXR), at 30 μM, induces functional expression of *ABCB1* in hPCISs over 24- and 48-h incubation (the time to allow complete induction to occur). Rifampicin significantly increased gene expression, protein levels, and efflux activity of ABCB1. Moreover, we described dynamic changes in ABCB1 transcript levels in hPCISs over 48 h incubation. We also observed that peaks of induction are achieved among donors at different times, and the extent of *ABCB1* gene induction is proportional to PXR mRNA levels in the intestine. In conclusion, we showed that hPCISs incubated in conditions comparable to those used for inhibition studies can be used to evaluate drugs’ ABCB1-inducing potency in the human intestine. Thus, hPCISs may be valuable experimental tools that can be prospectively used in complex experimental evaluation of drug–drug interactions.

## Introduction

P-glycoprotein (ABCB1) is an ATP-binding cassette transporter localized in the apical (lumen-facing) membrane of human enterocytes (Varma et al., 2005; Oostendorp et al., 2009; Giacomini and Huang, 2013). It controls cellular efflux into the intestinal lumen and thus governs the net intestinal uptake of its substrates, which include many xenobiotics. Thus, it provides important protection from absorption of potentially harmful ingested substances, but less helpfully, it may also block absorption of various drugs (Varma et al., 2005; Oostendorp et al., 2009; Giacomini and Huang, 2013). ABCB1 is also the site of clinically relevant drug–drug interactions (DDIs) (van Roon et al., 2005; Marchetti et al., 2007; Fenner et al., 2009; International Transporter Consortium, 2010; Giacomini and Huang, 2013). For example, inhibition of intestinal ABCB1 is responsible for increases in the bioavailability of dabigatran and digoxin (Su and Huang, 1996; Martin et al., 2015; Kumar et al., 2017), whereas its induction reportedly decreases the bioavailability of, for example, dabigatran, fexofenadine, digoxin, quinidine, and talinolol (Elmeliegy et al., 2020). Physiologically based pharmacokinetic modeling (PBPK) and clinical trials have also indicated possible DDIs (including reductions in intestinal drug absorption caused by ABCB1 induction) involving other drugs, such as tenofovir disoproxil fumarate, tenofovir alafenamide (Begley et al., 2018; Dhhs, 2019), bosutinib (Yamazaki et al., 2018), and nintedanib (Luedtke et al., 2018). In addition, many DDIs on intestinal ABCB1 may currently be missed, because *in vivo* testing does not readily distinguish between intestinal and hepatic contributions to the pre-systemic elimination (de Graaf et al., 2010; Groothuis and de Graaf, 2013). Because of the impact of DDIs on disposition of many drugs, these interactions should be investigated during both preclinical and clinical drug development according to the guidelines recommended by the U.S. Food and Drug Administration (FDA) in cooperation with the International Transporter Consortium, or the European Medicines Agency (EMA) (International Transporter Consortium, 2010; Giacomini and Huang, 2013; Giacomini et al., 2018; FDA, 2020).

Tests to assess drug-mediated inhibition of intestinal ABCB1 have been established (Guo et al., 2018; Martinec et al., 2019), but it does not hold true for induction (Elmeliegy et al., 2020; FDA, 2020). Due to limitations of available methods, the FDA currently does not recommend a specific *in vitro* approach for testing investigational drugs’ induction effects on transporters (FDA, 2020). This was also highlighted in the recent white paper published in 2021, where authors stated: “A validated *in vitro* system to study intestinal P-gp induction currently is not available and quantitative approach to predict the exposure of inducer drugs in the gut is still limited. Therefore, for foreseeable future determining definitively whether a drug induces intestinal P-gp and subsequent dosing recommendation will be based on clinical studies in conjunction with PBPK modeling and/or clinical induction calibration approaches” (Zamek-Gliszczynski et al., 2021). As a surrogate technique for predicting the pharmacokinetic effect of PXR-mediated ABCB1 induction *in vivo*, the FDA currently recommends using data from CYP3A induction studies as the ABCB1 levels correlate with magnitude of CYP3A induction (Lutz et al., 2018; FDA, 2020).

Studying the induction of intestinal ABCB1 directly in humans is technically and ethically challenging, and animal models have major flaws due to differences in induction cascades and characteristics of nuclear receptor ligands (Burger et al., 2005; Shirasaka et al., 2006; Rodrigues et al., 2009). So, drugs’ potential to induce intestinal ABCB1 is usually tested using cell lines, such as Caco-2 (Burger et al., 2005; Shirasaka et al., 2006; Rodrigues et al., 2009), LS180 (Weiss et al., 2009), or LS174T transfected with nuclear receptors (Cerveny et al., 2007; Sun et al., 2008; Smutny et al., 2013). However, expression patterns of transporters and transcription factors, including pregnane X receptor (PXR) and retinoid X receptor (RXR), are nonphysiological in these cell lines (Smutny et al., 2013; Negoro et al., 2016). Moreover, ABCB1 expression changes during the differentiation process (Goto et al., 2003), and between-lab variations in the abundance of transporters in the cells have been reported (Harwood et al., 2016). All these and other factors hinder the interpretation of data collected from cell lines and their extrapolation to situations *in vivo* (Elmeliegy et al., 2020). Human intestinal explants have also been described, but their morphology changes rapidly, and limited data are available on drug metabolism and transport (Groothuis and de Graaf, 2013). Fortunately, many drawbacks of intestinal explants can be avoided by preparing human precision-cut intestinal slices (hPCISs) (Groothuis and de Graaf, 2013). hPCISs are very thin (250–400 µm) sheets of intestinal mucosa prepared by slicing tissue embedded in low-gelling temperature agarose (de Graaf et al., 2010). Large numbers of slices, prepared from intestinal mucosa, can be obtained from human samples and used to analyze complex phenomena (Groothuis and de Graaf, 2013), including the toxicity and metabolism of drugs (van de Kerkhof et al., 2008; de Graaf et al., 2010). We previously used hPCISs to investigate the inhibitory effect of antiviral drugs on intestinal ABCB1 transporters (Martinec et al., 2019). Importantly, cells in hPCISs remain in their natural environment and retain physiological expression of drug-metabolizing enzymes, membrane transporters, and associated regulatory factors (van de Kerkhof et al., 2008; Groothuis and de Graaf, 2013). Moreover, it has been reported that 1-day incubation does not affect morphology and intracellular ATP levels, and exposure to rifampicin (RIF), a model ligand of PXR, increases mRNA levels of ABCB1 in jejunal hPCISs (van de Kerkhof et al., 2008).

The remaining questions, which are addressed here, are based on whether the reported increase in ABCB1 mRNA levels (van de Kerkhof et al., 2008) is reflected in elevated protein amount and efflux activity. Furthermore, it was studied whether morphology, ATP levels, and the induction are preserved after incubation up to 48 h. Moreover, we investigated the dynamics of changes in levels of ABCB1 transcripts in control and RIF-exposed hPCISs and the correlation between *PXR* gene expression and *ABCB1* induction.

## Materials and Methods

### Chemicals

Rhodamine 123 (RHD123), dimethyl sulfoxide (DMSO), acetonitrile (ACN), ethanol, low-gelling temperature agarose (type VII-A), amphotericin B solution (250 μg/ml), D-glucose, HEPES, RIF, sodium chloride, ethylenediaminetetraacetic acid (EDTA), formic acid, Tris-HCl, dichloromethane, sodium acetate, CP100356 [a model ABCB1 inhibitor (Martinec et al., 2019)], and bovine serum albumin (BSA) were purchased from Sigma-Aldrich (St. Louis, MO, United States). All chemicals were of at least analytical grade. William’s Medium E (WME) with Glutamax-I and gentamicin (50 μg/ml) solution was obtained from Thermo Fisher (Waltham, MA, United States). Formaldehyde solution (4%) was purchased from Mallinckrodt Baker B.V. (Deventer, Netherlands). Stock solutions of RIF and CP100356 used in all experiments were prepared in DMSO.

### Human Tissue Samples

Intestinal (jejunal) tissue samples were collected from eight patients (age range: 23–70 years) at the University Medical Center Groningen (UMCG, Netherlands) and four patients (age range 53–75) at the University Hospital Hradec Kralove (UHHK), Charles University (Czech Republic), during pylorus-preserving pancreaticoduodenectomy procedures performed for tumor in the head region. Tissue samples from Dutch patients were used for accumulation experiments and immunohistochemistry. Tissue samples from Czech patients were used for qRT-PCR. After resection, the tissue was immediately put in the ice-cold oxygenated Krebs–Henseleit buffer and transported to the laboratory within 15 min. All experiments were approved by local research ethics committees (UMCG and UHHK). All patients signed the informed consent for “further use” of coded-anonymous human tissue.

### Preparation and Incubation of hPCISs

hPCISs were prepared as previously described (de Graaf et al., 2010; Martinec et al., 2019). Briefly, the mucosa was separated from the muscular layer, dissected into fragments measuring approximately 5 by 20 mm, and then embedded in a low-gelling temperature agarose solution (3% w/v in 0.9% NaCl, 37°C). Approximately 300-µm-thick hPCISs were cut using a Krumdieck tissue slicer (Alabama R&D, Munford, AL, United States), then incubated in 12-well culture plates in 1.3 ml of WME, and supplemented with D-glucose (final concentration 25 mM), gentamicin (50 μg/ml), and amphotericin B (2.5 μg/ml) (Li et al., 2017a; Martinec et al., 2019). hPCISs were incubated in a humidified atmosphere of 80% O_2_ and 5% CO_2_ at 37°C with reciprocal shaking at approximately 90 cycles per minute. Medians of ATP levels in all analyzed hPCISs were above 5.3 pmol/μg, indicating good viability of tested samples.

### Accumulation of RHD123

To evaluate ABCB1 efflux activity we used a well-established method based on RHD123 uptake (Storch et al., 2007; Forster et al., 2012; Li et al., 2015, Li et al., 2017a; Martinec et al., 2019). Freshly prepared hPCISs and hPCISs incubated in WME for 24 or 48 h were preincubated for 30 min with and without the ABCB1 model inhibitor CP100356 (2 µM). Samples were transferred into the fresh WME incubation medium containing RHD123 (10 µM) with or without CP100356 (2 µM) (Martinec et al., 2019). Both preincubation and incubation steps were performed in a humidified atmosphere of 80% O_2_ and 5% CO_2_ at 37°C, with reciprocal shaking at approximately 90 cycles per minute (de Graaf et al., 2010; Li et al., 2017a; Martinec et al., 2019). Accumulation was halted after 2 h by washing the hPCISs twice with the cold (4°C) Krebs–Henseleit buffer. DMSO was added to all controls to the same final concentration as in CP100356-exposed hPCISs (0.2%). Samples were stored at −20°C prior to the RHD123 quantification, and samples for qRT-PCR and ATP analysis were stored at −80°C.

### ABCB1 Induction Study

RIF, a model inducer of ABCB1 and CYP3A4 (Wang and LeCluyse, 2003), was added to the culture medium of the hPCISs at the lowest concentration (30 µM), causing maximal inductive effect in both 24 and 48 h incubations (Supplementary Figure S1) (van de Kerkhof et al., 2008). hPCISs without RIF incubated in parallel were used as controls. Samples were collected after 24 and 48 h for immunohistochemical ABCB1 staining and after 4, 8, 12, 16, 20, 24, and 48 h for comparative qRT-PCR evaluation of ABCB1, CYP3A4, PXR, and RXRA mRNA levels in RIF-exposed hPCISs and corresponding controls. DMSO was added to all controls to the same final concentration as in RIF-exposed hPCISs (0.2%).

### Immunohistochemical Evaluation of ABCB1 Expression in the Intestinal Brush Border Layer

To assess the protein expression and localization of ABCB1 immunohistochemical staining, subsequent quantification was performed. hPCISs (*n* = 3 donors per condition) were fixed in 4% buffered formalin for 24 h and dehydrated in 70% ethanol for >24 h. After dehydration, the slices were embedded in paraffin, and then 4 μm thick sections were cut and placed on glass slides. Following deparaffinization and rehydration, antigens were retrieved by incubating the slides in Tris/EDTA buffer (pH 9.0) for 15 min near the boiling point. Nonspecific binding was blocked by incubating the slides in 2% BSA/2% rat serum in PBS at room temperature for 10 min with no antibody and then for a further hour with a primary antibody: recombinant anti-ABCB1 antibody (1:100; Abcam, Cambridge, United Kingdom, cat. no. ab170904). To block endogenous peroxidases, slides were incubated in 0.1% H_2_O_2_ for 15 min. Next, they were incubated at room temperature for 30 min with a secondary antibody (goat anti-rabbit horseradish peroxidase; 1:50; Dako, Glostrub, Denmark) in 2% BSA/2% human serum in PBS, and subsequently with a tertiary antibody (rabbit anti-goat horseradish peroxidase; 1:50; Dako, Gostrub, Denmark) in 2% BSA/2% human serum in PBS for 30 min at room temperature. To detect labeled antigens, an ImmPACT NovaRED kit (Vector Laboratories, Burlingame, United States) was used according to the manufacturer’s recommended protocol. Sections were counterstained with hematoxylin. Images were acquired using a Nanozoomer Digital Pathology Scanner (NDP Scan U10074-01, Hamamatsu Photonics K.K. Japan), and ABCB1 levels were quantified using Aperio ImageScope software (Leica Biosystems Imaging, Inc., United States) as previously described (Ruigrok et al., 2019).

### Analysis of Intracellular ATP and Protein Concentrations

The viability of the hPCISs was evaluated by intracellular ATP and protein concentration analyses as previously described (Li et al., 2017a; Martinec et al., 2019). ATP concentrations were measured, using a CLS II ATP bioluminescence assay kit (Roche, Mannheim, Germany), in hPCISs immediately following preparation and after both 24 and 48 h incubation, as well as in RIF-treated samples. ATP concentrations determined in hPCISs were normalized with respect to the protein content of pellets obtained during the process. For this purpose, they were dried overnight at 37°C and then solubilized in 200 μl of 5 M NaOH for 1 h at 37°C. Finally, Milli-Q water (800 μl) was added to the samples to give NaOH concentration of 1 M, and their protein contents were determined using a Lowry Protein Assay Kit (Bio-Rad DC Protein Assay, Veenendaal, Netherlands). The tissue with the ATP content above 1.5 nmol/mg of protein was considered viable (van de Kerkhof et al., 2008).

### Quantification of Gene Expression Levels

To obtain a sufficient quantity of RNA, three slices per donor from each condition were pooled, and total RNA was isolated using TRI Reagent (Molecular Research Center, Cincinnati, OH, United States) following the supplier’s recommended protocol. The integrity of the RNA samples was confirmed by electrophoretic separation on a 1.5% agarose gel, and total RNA concentrations were calculated from the A260 measurements. The purity of the isolated RNA was checked by measuring its A260/280 ratio using a NanoDrop 1000 spectrophotometer (Thermo Fisher Scientific, Delaware, United States). RNA (1 µg) was converted into cDNA in 20 µl reaction mixtures, using ProtoScript II Reverse Transcriptase (New England Biolabs, Ipswich, MA, United States) and a T100 thermal cycler (Bio-Rad Laboratories, Hercules, CA, United States), following the manufacturers’ recommendations. Portions of the cDNA (22.5 ng) were then amplified in a 384-well plate, with total reaction volumes of 5 µl per well, using the TaqMan Universal Master Mix II without uracil-DNA glycosylase and predesigned TaqMan Real-Time Expression PCR assays (Thermo Fisher Scientific, Waltham, MA, United States) for human *ABCB1* (Hs00184500_m1), *CYP3A4* (Hs00604506_m1), *PXR* (Nuclear Receptor Subfamily 1 Group I Member 2; Hs01114267_m1), and *RXRA* (Nuclear Receptor Subfamily 2 Group B Member 1; Hs01067640_m1). Expression levels of target genes were normalized with respect to the expression of *B2M* (Hs00984230_m1), after confirming the stability of the reference gene’s expression. Each sample was amplified in triplicate using the following PCR cycling profile: 95°C for 3 min, followed by 40 cycles at 95°C for 15 s and 60°C for 60 s. Relative expression levels were quantified using the ∆∆Ct method and arbitrary units (a.u.) were calculated as 2^∆Ct^ (with expression normalized to that of the housekeeping gene *B2M*) × 10^6^.

### RHD123 Quantification

The concentration of RHD123 in hPCISs was quantified with a Tecan Infinite 200 M plate reader (Tecan Group, Männedorf, Switzerland), using excitation and emission wavelengths of 485 and 528 nm, respectively, as previously described (Li et al., 2017a; Martinec et al., 2019). Before quantification, 600 µl of ACN:water solution (2:1) and approximately 300 mg of glass mini beads (diameter, 1.25–1.65 mm; Carl Roth, Karlsruhe, Germany) were added to each slice. PCIS were homogenized with a mini bead beater twice for 45 s. Samples were then centrifuged (10 min; 7,800 g), and the supernatant was collected for analysis. As RHD123 fluorescence can be quenched by other drugs (Storch et al., 2007), we analyzed quenching effect of increasing RIF concentration (up to 30 µM) on RHD123 fluorescence in ACN solution. We found that RIF quenched RHD123 (0.5 and 1.0 µM) fluorescence at concentrations higher than 10 and 20 μM, respectively (Supplementary Figure S2). As the concentration of RIF released from hPCISs into the ACN during sample preparation did not exceed 1 μM, a simple six-point calibration curve (0.000, 0.0625, 0.125, 0.250, 0.500, and 1.000 µM) without added RIF was used to determine RHD123 concentrations in hPCIS-based experiments. Concentrations of RHD123 were normalized against the protein content.

### Statistical Analyses

The statistical significance of between-treatment differences in ABCB1 activities determined in the human *ex vivo* transport experiments was assessed using the Wilcoxon signed-rank test. The significance of differences in measured ATP concentrations and RHD123 accumulation in fresh hPCISs and hPCISs after incubation for 24 or 48 h was assessed using the nonparametric Kruskal–Wallis analysis followed by Dunn’s test. One-sample *t*-tests were used to compare mean expression levels of focal genes at transcript or protein levels in samples from different donors. Differences were considered significant if *p* < 0.05. All data were processed using GraphPad Prism 8.31 (GraphPad Software, Inc., San Diego, CA, United States). Correlograms displaying Pearson’s correlation coefficient gene expression in hPCISs (considers all times points, in which gene expressions were analyzed) were generated using R-Studio 1.3.959.

## Results

### Morphology of the Intestinal Epithelial Cells Layer Remains Intact in hPCISs Incubated for 48 h

**FIGURE 1:**
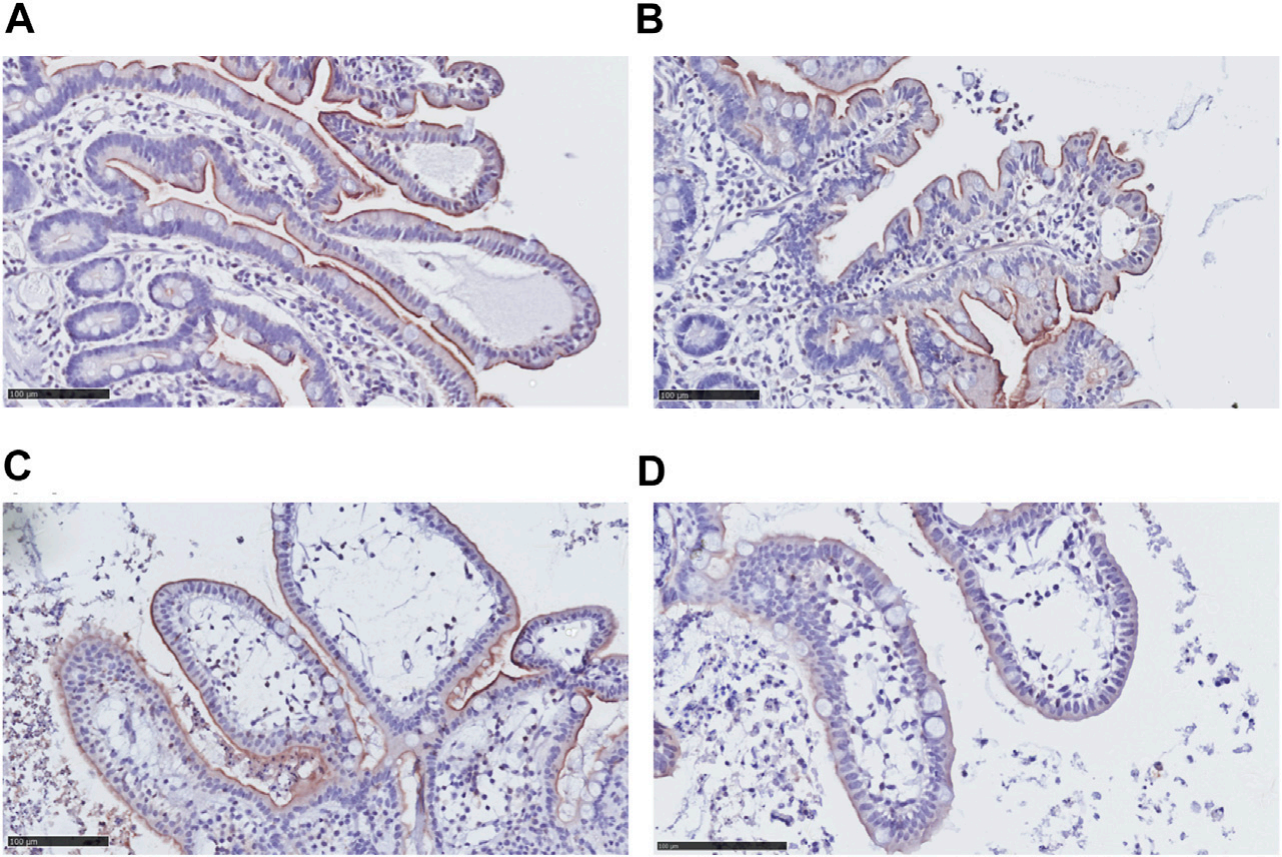
Morphology and ABCB1 expression in non-sliced intestine and hPCISs. Intact brush border epithelium and significant ABCB1 positivity (brown color) were observed in the non-sliced tissue **(A)** in fresh hPCISs, time = 0 h **(B)**, after 24 h **(C)**, and in hPCISs incubated for 48 h **(D)**. The slides were counterstained with hematoxylin (scale bar = 100 µm).

We investigated morphological changes of the villi and protein levels of ABCB1 in the epithelial cell layer in hPCISs for 48 h incubation. Extending a previous investigation of the condition of hPCISs after 24 h incubation (van de Kerkhof et al., 2008), we found that the epithelial cells in hPCISs preserved their columnar shape with no swelling or necrosis, and goblet cells remained intact (Figures 1A–D). ABCB1 staining (brown color) in villi was observed in non-sliced intact tissue, confirming the validity of the staining method (Figure 1A). Levels of ABCB1 were clearly detected in freshly prepared hPCISs (Figure 1B) and hPCISs incubated in the WME medium for both 24 h (Figure 1C) and 48 h (Figure 1D).

### ATP Levels Remain Stable in hPCISs Incubated for 48 h

**FIGURE 2:**
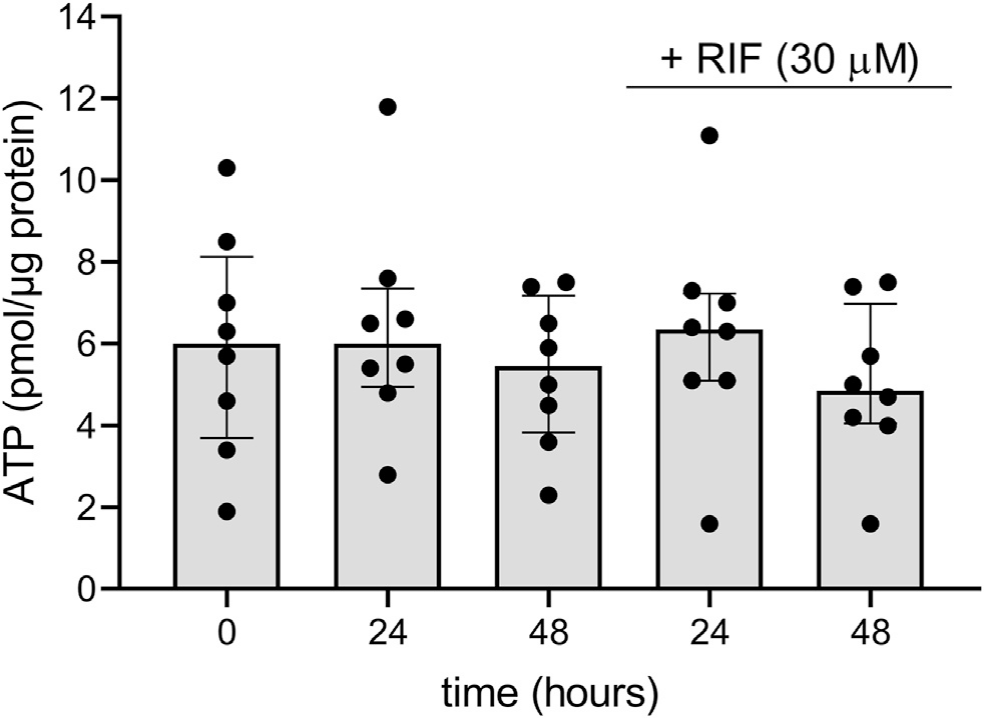
ATP contents in freshly prepared hPCISs (0 h) and after incubation in WME for 24 and 48 h with or without RIF (30 µM). Presented data are medians calculated from eight donors with interquartile ranges. Dots represent individual donor’s values. No significant differences between RIF-free and RIF-treated samples were detected by the nonparametric Kruskal–Wallis analysis followed by Dunn’s test.

To evaluate the viability of the hPCISs during the course of the incubations, we quantified their ATP content, which is an established marker of vital cellular processes (de Graaf et al., 2010; Li et al., 2017a; Martinec et al., 2019). The median of measured ATP concentrations in freshly prepared hPCISs (*n* = 8) was 6.0 pmol/μg protein. We detected comparable levels of ATP in hPCISs incubated in the WME medium for 24 and 48 h, 6.0 and 5.5 pmol/μg, respectively. We previously showed that neither solvent nor CP00356 affects ATP levels in hPCISs (Martinec et al., 2019), and in this study, we detected ATP levels of 6.4 and 4.9 pmol/μg protein in samples incubated with RIF (30 µM) for 24 and 48 h, respectively. There were no significant differences in the ATP content between any of the sets (*n* = 8) of tested samples (Figure 2), clearly suggesting that incubation in WME or WME with RIF (30 µM) for up to 48 h does not affect metabolic activity in hPCISs.

### Activity of ABCB1 Transporter Is Preserved in hPCISs Incubated for 48 h

**FIGURE 3:**
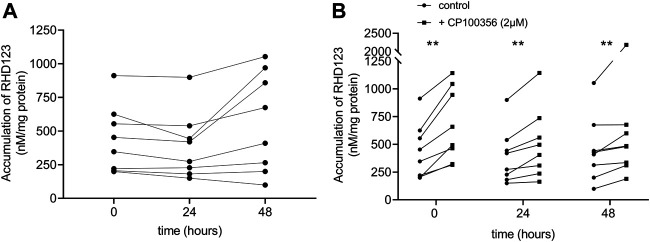
Accumulation of RHD123 analyzed in fresh hPCISs and hPCISs after incubation for 24 or 48 h. Each line represents samples collected from one donor. Corresponding calculated medians did not differ according to Kruskal–Wallis tests (*n* = 8) in the absence of CP100356 **(A)**. The addition of 2 µM CP100356 significantly increased the accumulation of RHD123 in hPCISs treated at all three time points (*n* = 8). Data are presented as a pairwise comparison showing individual donor values (control/CP100356-treated) together with indications of the significance of between-treatment differences according to the Wilcoxon signed-rank test (*, *p* < 0.05; **, *p* < 0.01) **(B)**.

To assess ABCB1 activity in hPCISs, we evaluated RHD123 accumulation in the hPCISs (initial concentration in the medium: 10 µM), as previously described (Storch et al., 2007; Li et al., 2015; Li et al., 2017a; Martinec et al., 2019). We found no significant difference in the uptake of RHD123 between freshly prepared hPCISs and hPCISs (*n* = 8) incubated in WME for 24 or 48 h (Figure 3A), and the medians of RHD123 concentrations were 539.1 nM/mg protein (*t* = 0 h), 346.6 nM/mg protein (*t* = 24 h), and 541.8 nM/mg protein (*t* = 48 h). Incubation in the presence of CP100356 (2 µM) significantly increased the RHD123 in all samples all tested times (Figure 3B; Supplementary Figure S3). These data strongly indicate that ABCB1 is active in hPCISs for at least 48 h.

### RIF Increases ABCB1 and CYP3A4 mRNA Levels in hPCISs Incubated for 48 h; Extent of Increase Correlates to *PXR* Gene Expression

**FIGURE 4:**
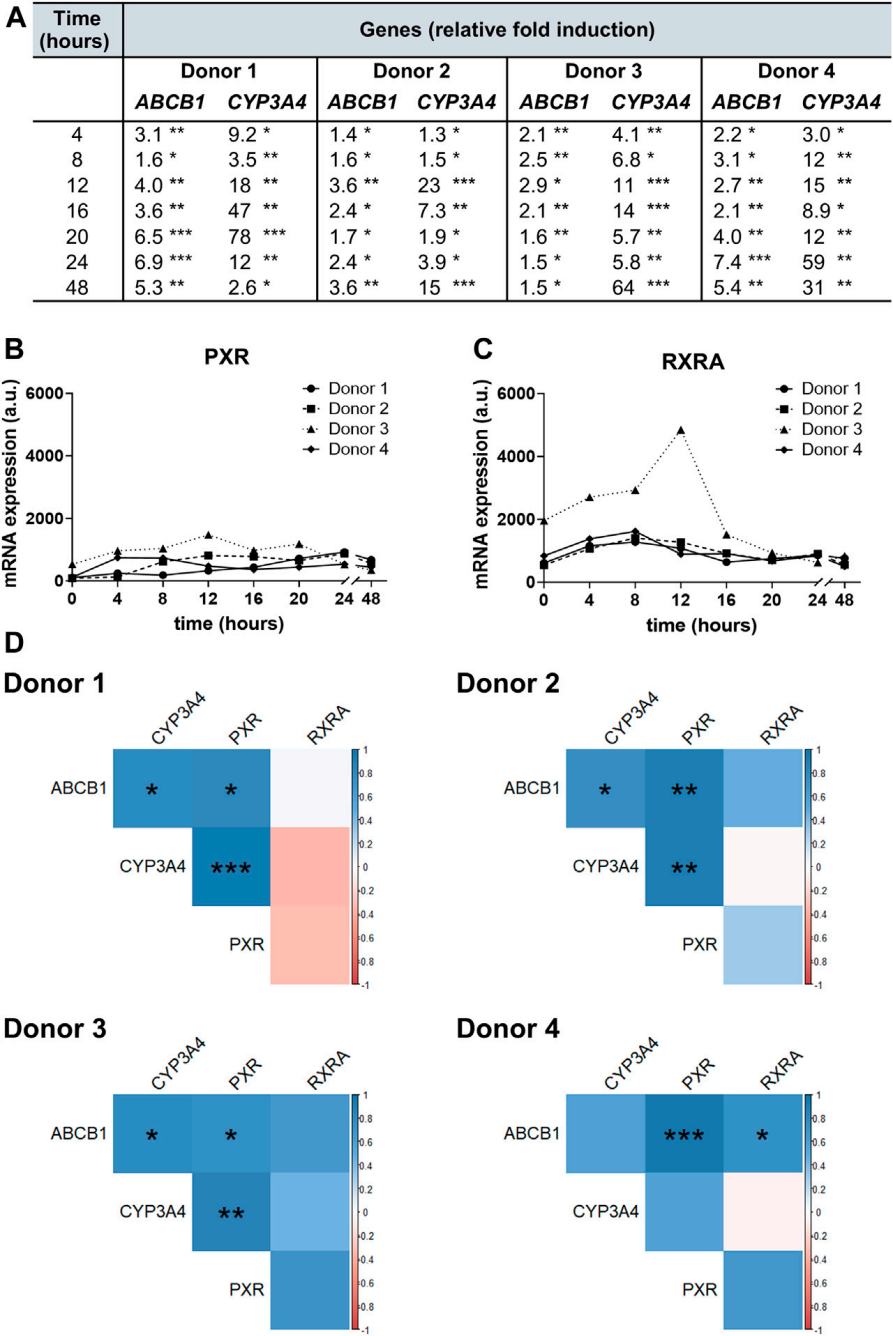
*ABCB1*, *CYP3A4*, *PXR*, and *RXRA* gene expression in hPCIS determined by qRT-PCR. Fold changes in ABCB1 and CYP3A4 mRNA levels in hPCISs incubated with RIF (30 µM) for 4, 8, 12, 16, 20, 24, and 48 h **(A)**, relative to expression in RIF-free controls, showing that RIF significantly increased mRNA levels of both genes in samples from all donors at all tested time points (one-sample *t*-tests, three slices from one donor: *, *p* < 0.05; **, *p* < 0.01; ***, *p* < 0.001). *PXR* **(B)** and *RXRA* **(C)** were demonstrated to be expressed in hPCISs incubated with RIF (30 µM) at all tested time points. Amounts of PXR and RXRA transcripts are presented in arbitrary units (a.u.), calculated as 2^−∆Ct^ × 10^6^. **(D)** The matrix of Pearson’s coefficients of correlation was used to assess associations among all targeted transcripts in RIF-treated hPCISs (positive in blue and negative in red, with color intensity proportional to the magnitude of the coefficients). Asterisks indicate significant differences: *, *p* < 0.05; **, *p* < 0.01; ***, *p* < 0.001.

To investigate RIF-mediated gene induction, we quantified *ABCB1* and *CYP3A4* gene expression in hPCIS samples obtained from each donor from the UHHK and compared fold changes in their expression between hPCISs incubated with RIF (30 µM) for 4, 8, 12, 16, 20, 24, and 48 h, and corresponding RIF-free controls. Levels of ABCB1 and CYP3A4 in control hPCISs did not change significantly during incubation (Supplementary Figures S4, S5). RIF induced *ABCB1* in all tested cases (up to 7.4-fold, in samples from donor 4 after 24 h incubation). Induction of *CYP3A4*, a gene highly inducible by RIF, was more pronounced: up to 78-fold in samples from donor 1 after 20 h incubation (Figure 4A). We also observed interindividual variability in basal and induced expression levels of these genes and peak induction occurring in the different donors at different time points (Supplementary Figures S4, S5). PXR and RXRA mRNA expression fluctuated over 48 h incubation in both RIF (30 µM)-treated (Figures 4B,C) and control hPCISs (Supplementary Figure S6). Higher RXRA transcript levels were observed after exposure to RIF in samples from donor 3 than in samples from other donors (Figure 4C), but basal RXRA mRNA levels in this donor were also higher (Supplementay Figure S6). Pearson’s correlation coefficient showed that expressions of both *ABCB1* (4/4 donors) and *CYP3A4* (3/4 donors) proportionally related to levels of PXR mRNA, and there was also a positive association between the amount of ABCB1 and CYP3A4 transcripts (3/4 donors) in RIF-treated hPCISs. ABCB1 levels also correlated with the expression of the *RXRA* gene in donor 4 (Figure 4D).

### RIF Increases Protein Level of ABCB1 in hPCISs Incubated for 48 h

**FIGURE 5:**
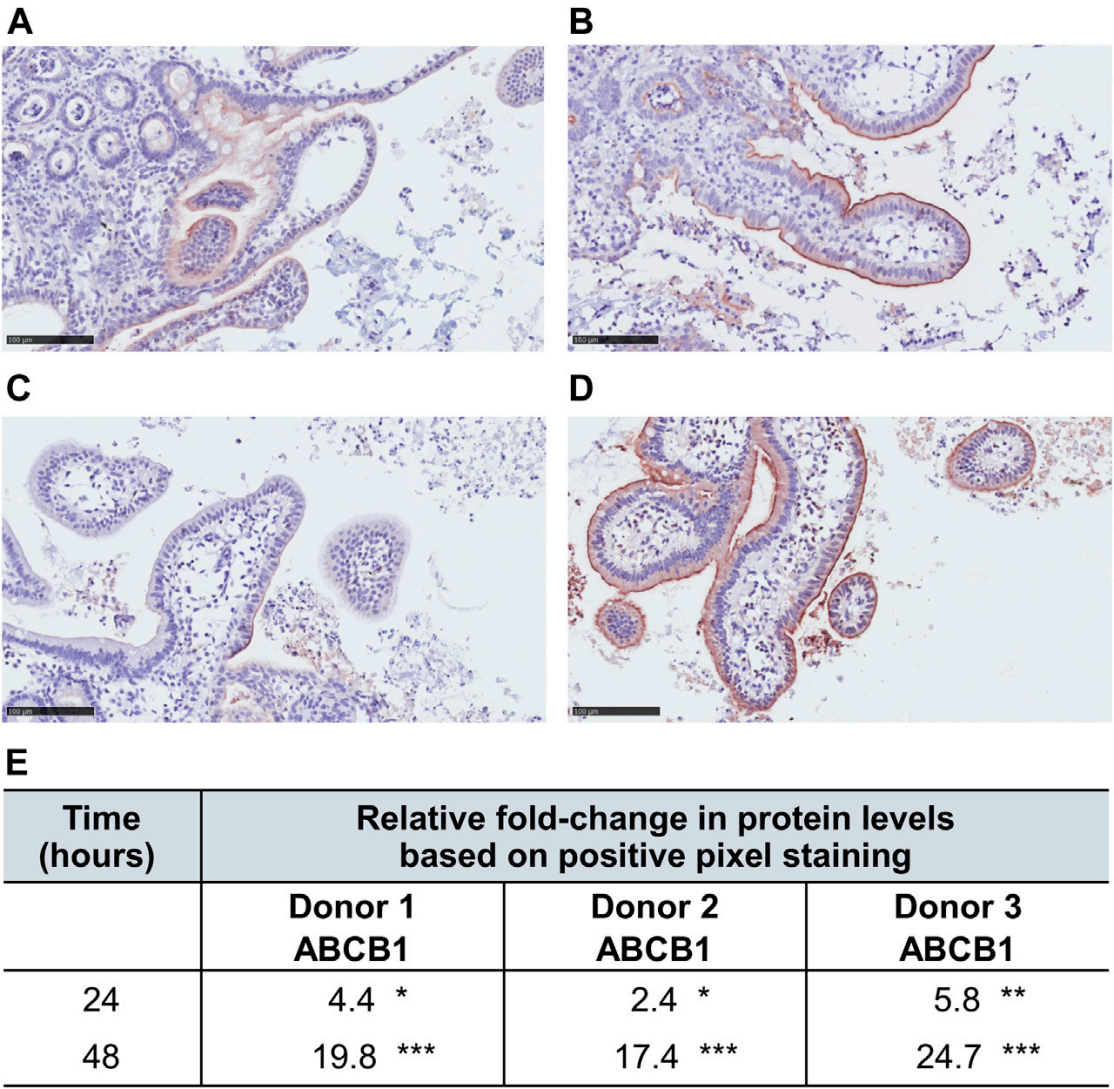
ABCB1 levels (indicated by brown staining) in RIF-free samples incubated for 24 h **(A)** and 48 h **(C)** and in counterparts treated with 30 µM RIF for 24 h **(B)** and 48 h **(D)**. The slides were counterstained with hematoxylin (scale bar = 100 µm). Panels **(A–D)** represent only a partial cutout of the whole section to demonstrate immunohistochemical staining. Images of the entire sections were used for the analysis. **(E)** Fold changes in numbers of strong ABCB1-positive pixels between images of RIF-treated and RIF-free hPCISs from donors after incubation for indicated times according to subsequent algorithmic analysis. Asterisks indicate significant differences between them according to one-sample *t*-tests (*n* = 3): *, *p* < 0.05; **, *p* < 0.01; ***, *p* < 0.001.

To confirm *ABCB1* upregulation by RIF at the protein level, we compared results of the immunohistochemical staining of hPCISs incubated for 24 and 48 h with and without 30 µM RIF. We observed decreased protein levels of ABCB1 in control hPCISs after 24 and 48 h when compared with freshly prepared hPCISs, but significant increase was observed in RIF-treated hPCISs in all three donors (Figure 5; Supplementary Figure S7). Relative increases in ABCB1 calculated as ABCB1 in RIF-treated divided by control hPCISs (both after 24 h incubation) were 4.4, 2.4, and 5.8 and in RIF-treated divided by control hPCISs (both after 48 h incubation) were 20, 17, and 25 (Figure 5E). When compared with freshly prepared hPCISs, we found 2.1-, 1.1-, and 1.4-fold increases for 24 h incubation and 12.1-, 2.1-, and 4.3-fold increases for 48 h (Supplementary Figure S7). Our data thus confirm that the RIF-mediated increases in ABCB1 mRNA levels are followed by increases in ABCB1 protein levels.

### RIF Increases Efflux Activity of ABCB1 in hPCISs Incubated for 24 and 48 h

**FIGURE 6:**
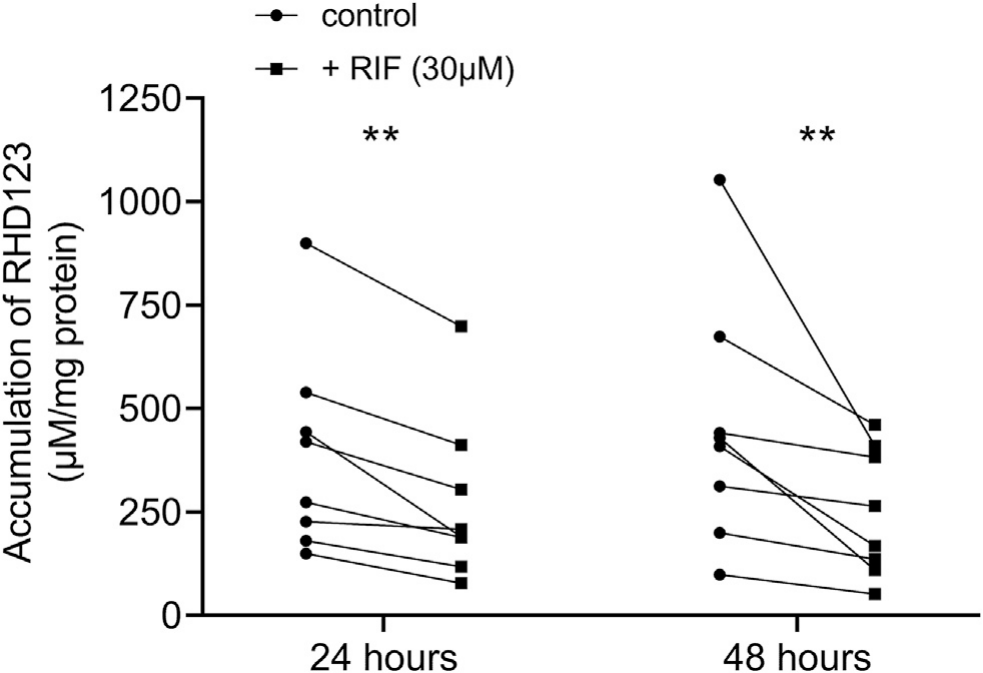
Levels of RHD123 accumulation in hPCISs incubated with 30 µM RIF were significantly lower than those in RIF-free samples after both 24- and 48-h incubations (Wilcoxon signed-rank tests: *, *p* < 0.05, and **, *p* < 0.01). Data are presented as pairwise comparisons between RIF-free (dot) and RIF-treated (square) samples. Each line represents an individual donor.

To confirm that the induction of ABCB1 in hPCISs incubated for 24 and 48 h with RIF (30 µM) is reflected in increased activity of ABCB1, we assessed RDH123 accumulation tested at 10 µM. At both time points, RIF significantly decreased levels of RHD123 in hPCISs in all eight donors in both tested time points (Figure 6; Supplementary Figure S8). Thus, RIF clearly increased ABCB1 efflux activity in hPCISs.

## Discussion

Current worldwide consumption of medication is enormous and steadily increasing, thereby raising risks of DDIs that were not detected during preclinical and clinical trials (Sandson, 2005; Seden et al., 2009; Evans-Jones et al., 2010). The most frequently used route of administration of a drug is oral ingestion. This route is considered convenient and the safest. However, after oral administration, intestinal absorption is affected by pre-systemic elimination that may involve ABCB1 and CYP3A4 activities. Induction of intestinal ABCB1 has been suggested to considerably decrease the bioavailability of drugs after oral administration (Elmeliegy et al., 2020). Therefore, according to the drug interaction guidelines, a drug’s potency to induce drug-metabolizing enzymes and transporters at the mRNA level should be investigated before drugs are commercially launched (Cole et al., 2020). Not only *ABCB1* can be strongly induced in enterocytes but also *CYP3A4*, mainly *via* the PXR pathway (Cerveny et al., 2007; van de Kerkhof et al., 2008).

Recently, Lutz et al. published data establishing proof of concept that induction of ABCB1 *in vivo* can be extrapolated based on analysis of CYP3A induction magnitude by PXR ligands (Lutz et al., 2018). However, as of yet, there is no widely accepted experimental model to study induction of metabolizing enzymes and transporters in the intestine that has high screening capacity and sufficiently similar characteristics to *in vivo* systems (Zamek-Gliszczynski et al., 2021). In this study, we used hPCISs prepared from the jejunum, which is the major site of drug absorption after oral administration (Murakami, 2017), and, moreover, it reveals a significant ABCB1 activity (Li et al., 2017a). Extending a previous study (van de Kerkhof et al., 2008), we tested the possibility of incubating hPCISs for up to 48 h. We detected no significant differences in morphology and ATP levels between hPCISs incubated for 48 h with and without RIF (30 µM) relative to freshly prepared hPCISs, and the observed characteristics were comparable to those reported for hPCISs incubated for 24 h (van de Kerkhof et al., 2008). ABCB1 levels were preserved in all analyzed hPCISs, and the activity of ABCB1 was not affected by the length of incubation between 0 and 48 h. Incubation with 2 µM CP100356 increased RHD123 accumulation 1.6-fold in fresh hPCISs, indicating inhibition of ABCB1 activity, which is in accordance with our previous findings (Li et al., 2017b; Martinec et al., 2019). The increase in RHD123 accumulation in CP100356-treated PCIS should be associated directly with ABCB1 inhibition because another important intestinal efflux transporter breast cancer resistance protein does not recognize RHD123 as a substrate (Honjo et al., 2001; Kalgutkar et al., 2009). As there were comparable increases in hPCISs incubated with CP100356 up to 48 h, we conclude that the ABCB1 activity is fully preserved during the incubation.

In this study, both ABCB1 and CYP3A4 were significantly increased in RIF-treated slices from each donor than in RIF-free counterparts at both transcript (Figure 4A) and protein (ABCB1) levels (Figure 5; Supplementary Figure S7). We observed higher relative increases in hPCISs after 48 h incubation (Figure 5E) than with the fold change (2–4) suggested by *in vivo* studies in duodenal biopsies (Greiner et al., 1999; Westphal et al., 2000) and PBPK modeling (Yamazaki et al., 2019). It was likely due to the decline in ABCB1 levels in control hPCISs over the course of incubation in the WME medium. As DMSO did not affect ABCB1 mRNA in control PCIS, it can be concluded that the observed decline in ABCB1 protein is due to the decreased stability of ABCB1. DMSO can be responsible for this phenomenon as it was suggested to destabilize some proteins and affect their binding properties (Tjernberg et al., 2006). Independently of DMSO, it can be caused by accelerated processes associated with ABCB1 degradation (Pokharel et al., 2017) and/or decreased activity of mechanisms that prevent ABCB1 proteolysis (Katayama et al., 2016). However, the decreasing effect is obviously overwhelmed by RIF-mediated induction. When compared with freshly prepared hPCISs, RIF elicited 1.1- to 2.1-fold increase in hPCISs incubated for 24 h and 2.1- to 12.1-fold increase in hPCISs incubated for 48 h. The observed increase higher than four is also likely because we used hPCISs prepared from proximal jejunum, while previous analyses of ABCB1 were performed in biopsies of duodenum (Greiner et al., 1999; Westphal et al., 2000), and we quantified ABCB1 protein using image analysis of immunohistochemical staining. This method is considered to be less precise than Western blot due to potential involvement of nonspecific signal into quantification.

In the RIF-treated slices, there was also a significant increase in the ABCB1 activity (Figure 6). This shows that RIF was able to induce not only ABCB1 on the gene and protein levels but also the ABCB1 activity. However, RIF might also decrease levels of influx organic cation transporter 1 (Han et al., 2013; Jouan et al., 2014) in the intestine as previously reported for hepatic cells (Hyrsova et al., 2016). Therefore, it cannot be ruled out that this phenomenon contributes to decreased accumulation of RHD123 in PCIS. RHD123 is also transported by influx organic anion transporter peptide 1A2. Contamination of our results by the activity of OATP1A2 can be, however, considered marginal because this transporter has only negligible intestinal expression (Oswald, 2019) and RIF does not seem to inhibit or downregulate it (Kalliokoski and Niemi, 2009).

To address the pathways involved in the induction of *ABCB1*, we studied PXR and RXR, as their heterodimer formation is a crucial step in the *ABCB1* and *CYP3A4* transactivation process in the presence of RIF (Li et al., 2017a; Martinec et al., 2019). We evaluated levels of PXR mRNA and the most abundantly expressed isoform of *RXR*, RXRA, in the human jejunum (Wang et al., 2005) and found that their transcript levels were maintained throughout the 0–48 h incubations (Figure 4; Supplementary Figure S6). Moreover, we here observed that the extent of *ABCB1* and *CYP3A4* induction correlates to PXR mRNA levels in hPCISs treated with RIF at all the time points tested (Figure 4D), which is in accordance with recent findings by Fritz et al. (2019). The correlation between PXR mRNA and/or protein and *ABCB1* gene expression was evidenced also in other tissues including the lungs, liver, blood, and mononuclear cells (Owen et al., 2004; Albermann et al., 2005; Kong et al., 2016; Wu et al., 2016). Moreover, we found a proportional association between induction of *ABCB1* and *CYP3A4*, which is in line with previous reports documenting shared regulation and interplay in the intestinal absorption (Wei et al., 2013).

Despite the indisputable advantages of hPCISs, they also have some drawbacks for evaluating drugs’ *ABCB1*-induction potency. Tight cooperation with a surgery department is required, and tests should be performed on tissue unaffected by tumor or other pathologies such as Crohn’s disease or ulcerative colitis. Considering this fact, pancreaticoduodenectomy, surgery providing the jejunal segment that is not related to intestinal disease, used in this study seems to be the optimal procedure. Moreover, interindividual differences in the expression of *ABCB1* and *CYP3A4* related to polymorphisms, the presence of inducing and inhibitory substances (from the diet or medication), patients’ gender and age, patients’ disease, and/or activities of nuclear factors (Lamba et al., 2002; Lindell et al., 2003; Siegmund et al., 2003; Groothuis and de Graaf, 2013) may complicate interpretation of results obtained from different laboratories. Perhaps, this phenomenon is reflected in the high variations among donors’ samples that we have seen in this study. Regarding device requirements, a laboratory has to be equipped with Krumdieck tissue slicer and an incubator with high oxygen content.

Our study has certain limitations. We did not correlate mRNA, protein levels, and ABCB1 function as their quantification was performed in different patients. The study was carried out in a small cohort of donors and data are, due to considerable interindividual variability, presented separately for each patient. Small cohorts and limited amount of provided intestinal tissue also made it impossible to determine EC_50_ and E_max_.

## Conclusion

Evaluation of a new model to test potency of drugs to induce intestinal ABCB1 is a long process. It requires testing multiple inductors together with various substrates including clinically relevant drugs and obtained results should be compared with those collected using the currently established methods. In this study, we took the first step to evaluate hPCISs as a valuable and more complex model to study intestinal ABCB1 induction. We demonstrated that hPCISs remain viable, intact, express ABCB1, CYP3A4, PXR, and RXRA mRNA, and preserve stable ABCB1 protein levels and function for 48 h incubation in the applied conditions. *ABCB1* functional expression and CYP3A4 levels are responsive to co-incubation with the PXR ligand, RIF, and the extent of *ABCB1* and *CYP3A4* induction is proportional to PXR mRNA levels. Moreover, we described dynamic changes in ABCB1 and CYP3A4 transcript levels in hPCISs over 48 h incubation, showing that peaks of induction are achieved among donors at different times. From the prospective view, hPCISs should be of interest to researchers involved in preclinical research and the pharmaceutical industry as data reported here and elsewhere (van de Kerkhof et al., 2008; Martinec et al., 2019) demonstrate their utility for *in vitro* evaluation of complex DDIs, involving both inhibition and induction of ABCB1 and other molecules, such as CYP3A4 and phase II metabolizing enzymes. Thus, they may help efforts to elucidate in detail the interplay of such pharmacokinetic factors in pre-systemic drug elimination.

## Data Availability

The raw data supporting the conclusions of this article will be made available by the authors, without undue reservation.

## Abbreviations

ABCB1, p-glycoprotein; ACE, accumulation experiments; ACN, acetonitrile; a.u., arbitrary unit; BSA, bovine serum albumin; CYP, Cytochrome P450; DDI, drug–drug interaction; DMSO, dimethyl sulfoxide; EDTA, ethylenediaminetetraacetic acid; EMA, European Medicines Agency; FDA, U.S. Food and Drug Administration; qRT-PCR, quantitative reverse-transcription polymerase chain reaction; hPCISs, human precision-cut intestinal slices; IHC, immunohistochemistry; PBPK, physiologically based pharmacokinetic modeling; PXR, pregnane X receptor; RHD123, rhodamine 123; RIF, rifampicin; RXRA, retinoid X receptor alpha; UHHK, the University Hospital Hradec Kralove; UMCG, the University Medical Center Groningen; WME, William’s medium E.
