# Supplementary material for: Rifampicin Induces Gene, Protein, and Activity of P-Glycoprotein (ABCB1) in Human Precision-Cut Intestinal Slices
Source: Front Pharmacol. 2021 Jun 9;12:684156. doi: 10.3389/fphar.2021.684156 (PMC8220149; doi:10.3389/fphar.2021.684156)
Supplement: Supplementary file 1 [file DataSheet1.pdf]

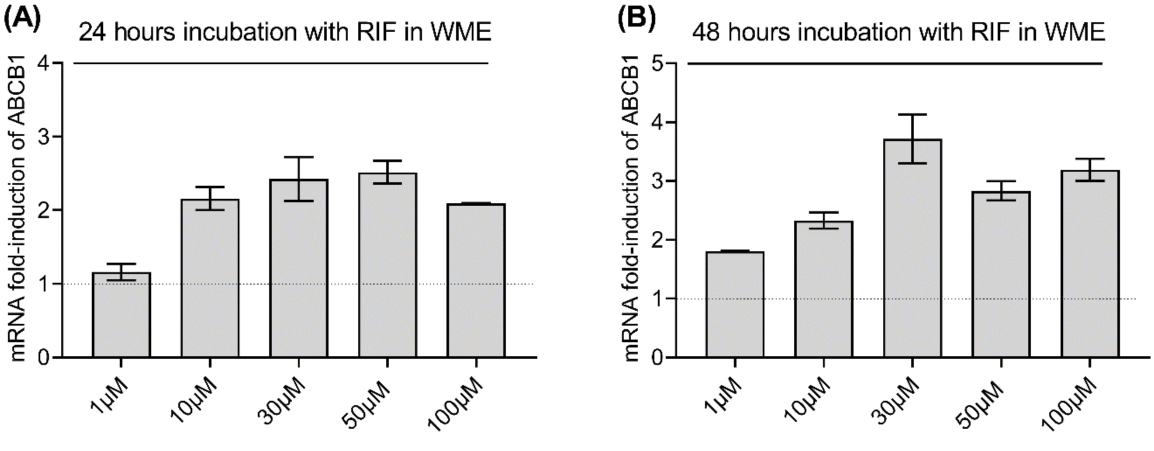

Figure S1. Effect of increasing concentrations of RIF on ABCB1 mRNA after 24 (A) and 48 (B) hours. RIF (30 µM) had the most profound effect. Presented data are means ± SD of relative gene expression levels in hPCISs prepared from a single donor. Control is shown as a dotted line.

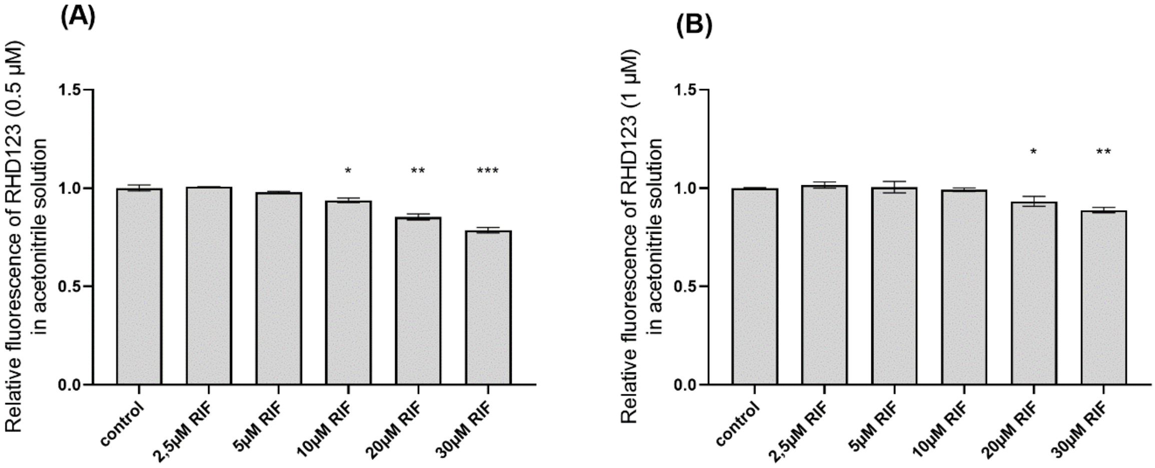

Figure S2. Quenching effects of selected concentrations of RIF on fluorescence of RHD123 (1 and 0.5 µM) in acetonitrile solution, with indications of significant differences according to one-way ANOVA followed by the Dunnett *post hoc* test: *, $p < 0.05$; **, $p < 0.01$; ***, $p<0.001$. Presented data are means ± SD (n = 3).

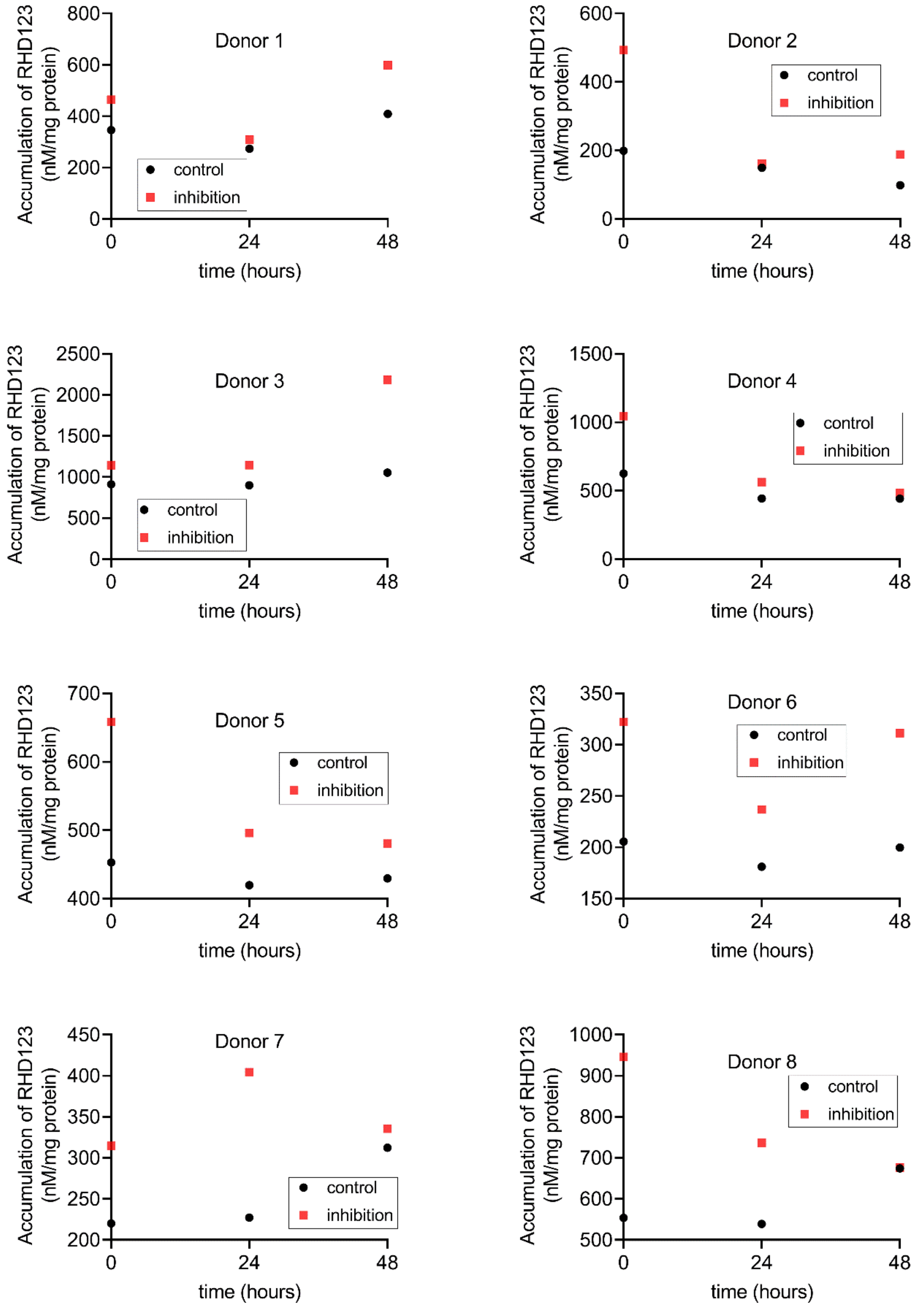

Figure S3. Accumulation of RHD123 in fresh hPCISs and hPCISs after incubation for 24 or 48 hours in the individual donors in presence or absence of the CP100356 inhibitor (2 µM).

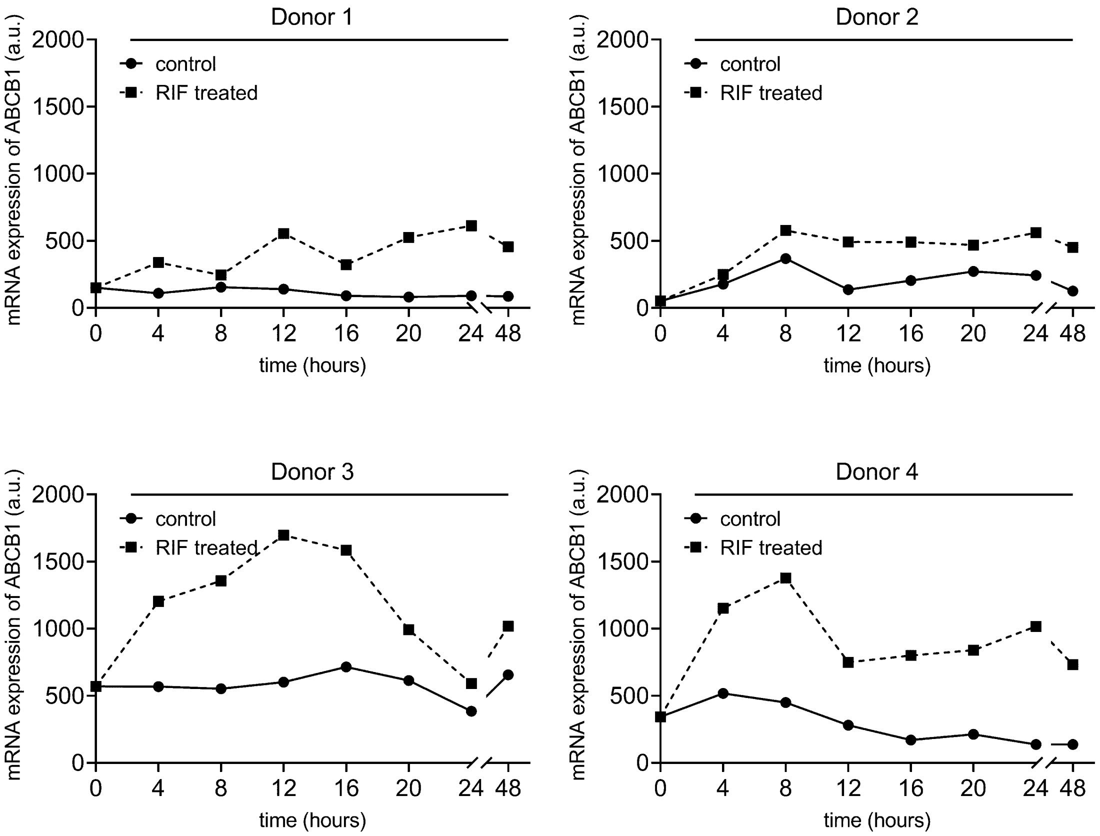

Figure S4. Changes in ABCB1 mRNA levels during 48 hours incubation in control and RIF-treated hPCISs. Increased levels of ABCB1 in hPCISs incubated with RIF (30 μM) were observed in samples from all donors at all selected time points. Presented data are means (from technical triplicates) of arbitrary units (a.u.) calculated as $2^{-\Delta Ct}$ (expression normalized to that of the housekeeping gene *B2M*) × 10^6.

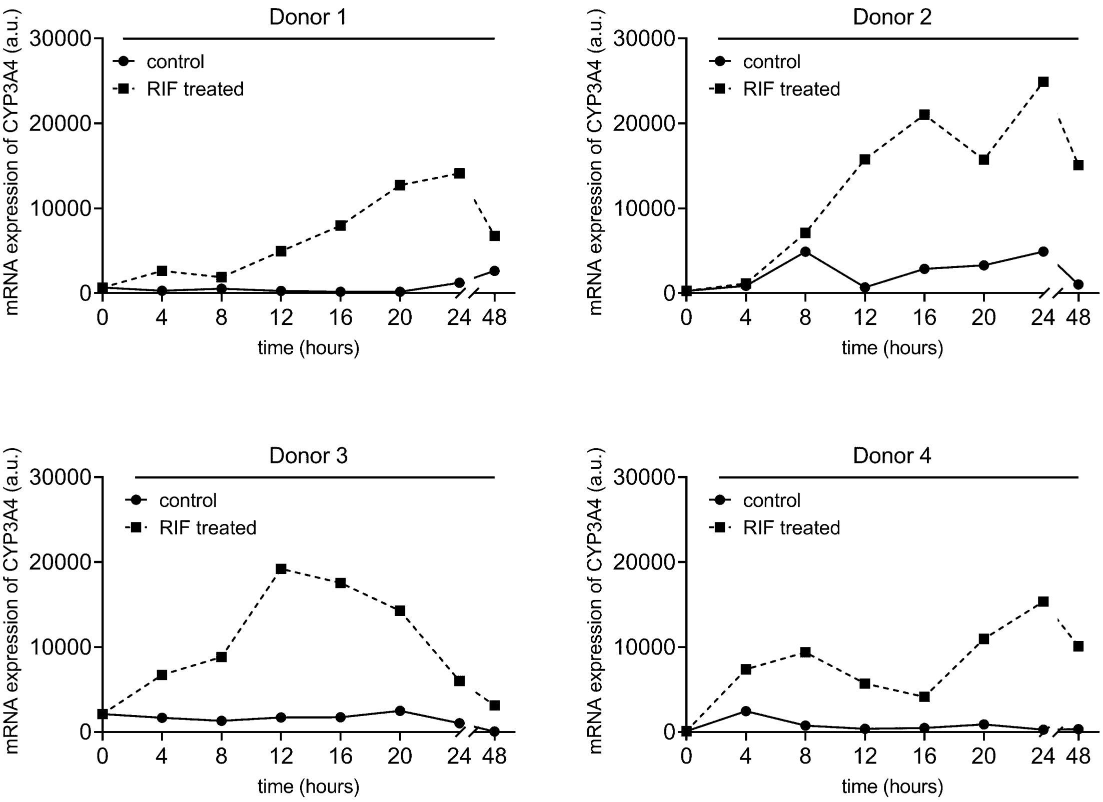

Figure S5. CYP3A4 mRNA levels during 48 hours incubation in control and RIF-treated hPCISs. Increased levels of CYP3A4 in hPCISs incubated with RIF (30 μM) was observed at all selected time points. Presented data are means (from technical triplicates) of arbitrary units (a.u.) calculated as $2^{-\Delta Ct}$ (expression normalized to that of the housekeeping gene *B2M*) × 10^6.

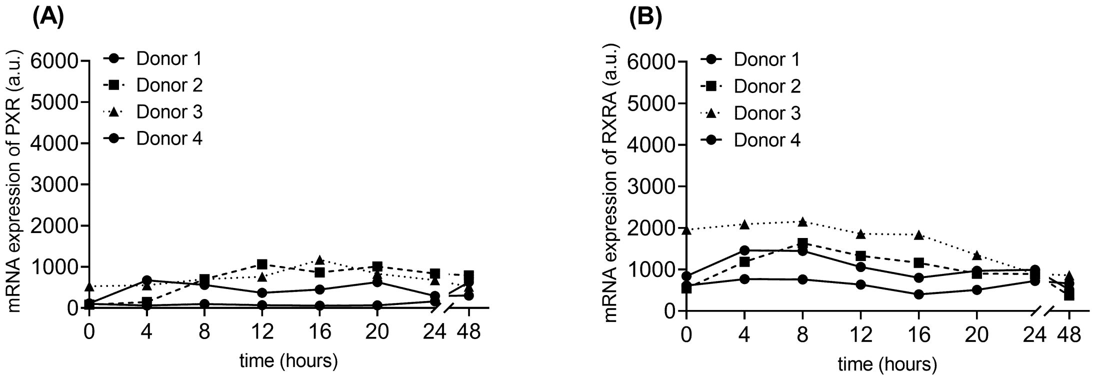

Figure S6. PXR (A) and RXRA (B) mRNA levels during 48 hours incubation in control hPCISs. Presented data are means (from technical triplicates) of arbitrary units (a.u.) calculated as $2^{-\Delta Ct}$ (expression normalized to housekeeping gene *B2M*) × 10^6.

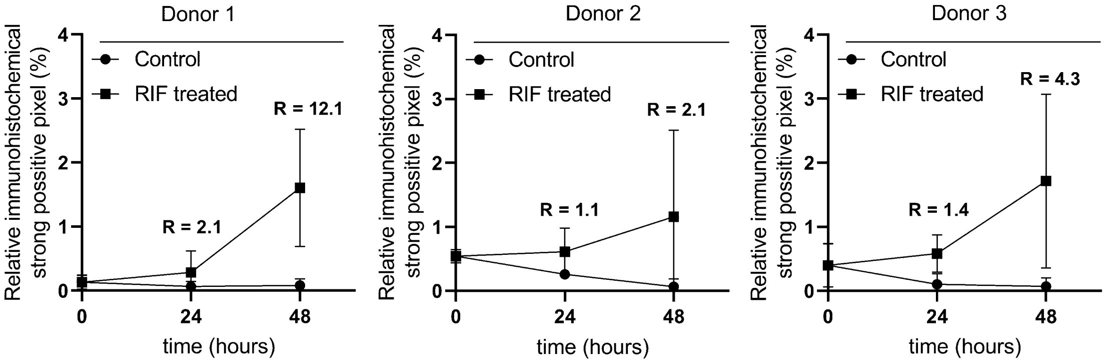

Figure S7. Changes of ABCB1-positive pixels between immunohistochemical images of RIF-treated and RIF-free hPCISs samples for individual donors. R = ratio of RIF-treated hPCISs (24 hours) or RIF-treated hPCISs (48 hours) divided by amount of ABCB1 in freshly-prepared hPCISs.

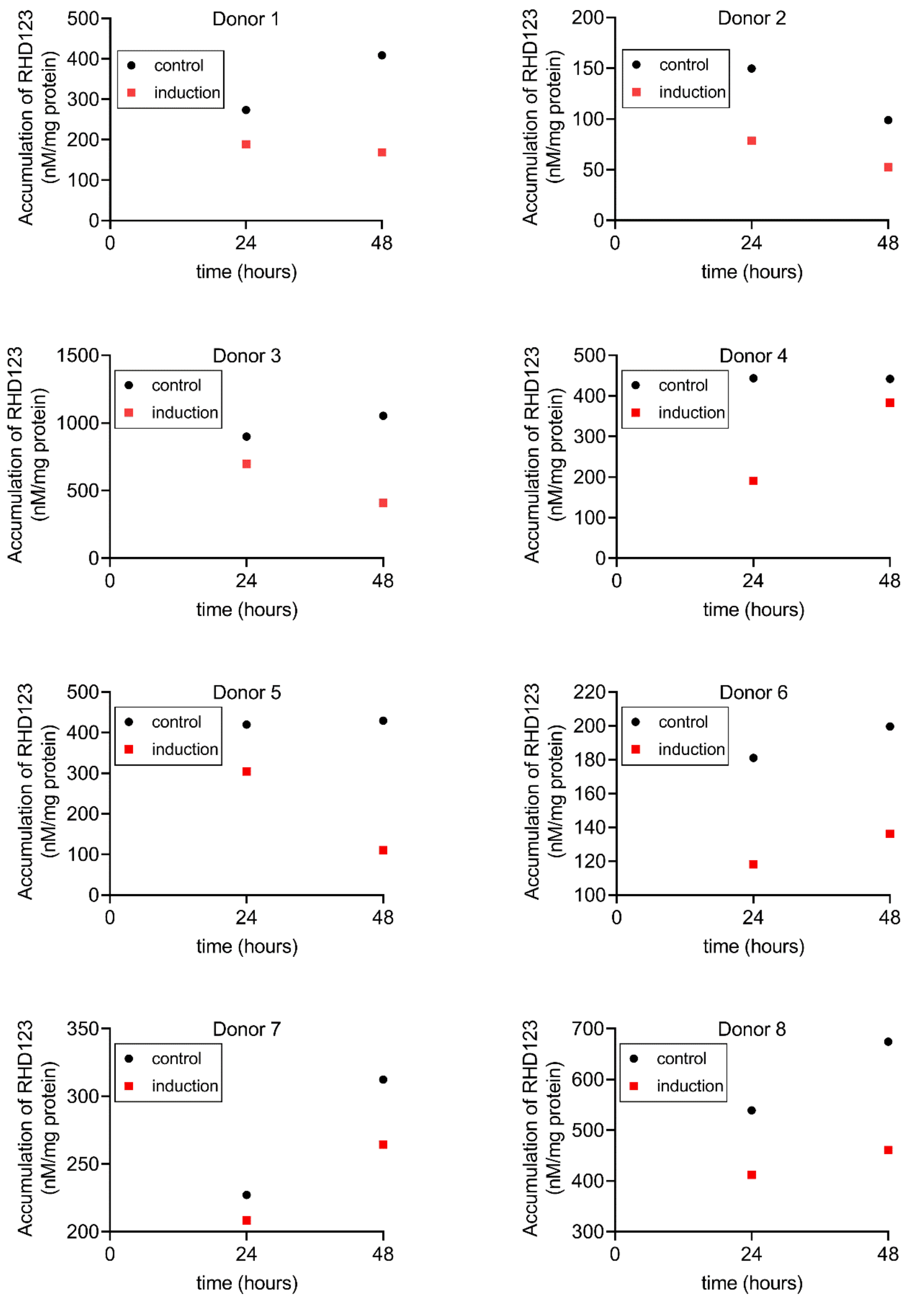

Figure S8. Levels of RHD123 accumulation in RIF treated (induction, 30 µM) and RIF-free (control samples) hPCISs after both 24 and 48 hours incubation in the individual donors.
